# Effects of aromatase inhibitor therapy on adiposity and cardiometabolic health in postmenopausal women: a controlled cohort extension study

**DOI:** 10.1530/EC-23-0076

**Published:** 2023-09-06

**Authors:** Yee-Ming M Cheung, Rudolf Hoermann, Karen Van, Damian Wu, Jenny Healy, Bella Halim, Manjri Raval, Maria McGill, Ali Al-Fiadh, Michael Chao, Shane White, Belinda Yeo, Jeffrey D Zajac, Mathis Grossmann

**Affiliations:** 1 Department of Medicine, The University of Melbourne, Austin Health, Melbourne, Australia; 2 Department of Endocrinology, Austin Health, Melbourne, Australia; 3 Division of Endocrinology, Diabetes and Metabolism, Northwell, Great Neck, New York, USA; 4 Department of Radiology, Austin Health, Melbourne, Australia; 5 Department of Cardiology, Austin Health, Melbourne Australia; 6 Olivia Newton-John Cancer Research and Wellness Centre, Austin Health, Melbourne, Australia; 7 Olivia Newton-John Cancer Research Institute, Austin Health, Melbourne, Australia

**Keywords:** aromatase inhibitor, breast cancer, cardiometabolic risk, visceral adipose tissue, visceral fat

## Abstract

**Purpose:**

We previously demonstrated that 12 months of aromatase inhibitor (AI) treatment was not associated with a difference in body composition or other markers of cardiometabolic health when compared to controls. Here we report on the pre-planned extension of the study. The pre-specified primary hypothesis was that AI therapy for 24 months would lead to increased visceral adipose tissue (VAT) area when compared to controls.

**Methods:**

We completed a 12-month extension to our prospective 12-month cohort study of 52 women commencing AI treatment (median age 64.5 years) and 52 women with breast pathology not requiring endocrine therapy (63.5 years). Our primary outcome of interest was VAT area. Secondary and exploratory outcomes included other measures of body composition, hepatic steatosis, measures of atherosclerosis and vascular reactivity. Using mixed models and the addition of a fourth time point, we increased the number of study observations by 79 and were able to rigorously determine the treatment effect.

**Results:**

Among study completers (AI = 39, controls = 40), VAT area was comparable between groups over 24 months, the mean-adjusted difference was −1.54 cm^2^ (95% CI: −14.9; 11.9, *P* = 0.79). Both groups demonstrated parallel and continuous increases in VAT area over the observation period that did not diverge or change between groups. No statistically significant difference in our secondary and exploratory outcomes was observed between groups.

**Conclusions:**

While these findings provide reassurance that short-to-medium-term exposure to AI therapy is not associated with metabolically adverse changes when compared to controls, risk evolution should be less focussed on the AI-associated effect and more on the general development of cardiovascular risk over time.

## Introduction

In postmenopausal women with early oestrogen receptor-positive (ER+) breast cancer, aromatase inhibitors (AIs) are the mainstay of adjuvant therapy (1). In this population, AI therapy is directed at the oestrogen axis and inhibits aromatase, the enzyme required for the conversion of androgens to oestradiol, causing effectively complete circulating oestradiol deficiency (2). In general, 5 years of AI adjuvant therapy is considered as a first-line treatment in women with early or locally advanced ER+ breast cancer (3). Treatment can be extended by a further 2–5 years in select populations thought to be at increased risk of breast cancer recurrence (4).

In women with early breast cancer, one of the most common causes of mortality is death from cardiovascular disease (CVD) (5). The mechanism for this increased mortality is likely multifactorial and includes the higher prevalence of CVD risk factors in cancer survivors and the cardiotoxic effects of breast cancer treatments, specifically radiotherapy and chemotherapy (6). Existing studies investigating the association between AI therapy and CVD mortality in primarily postmenopausal women have reported conflicting results (7, 8, 9, 10, 11, 12, 13, 14), with some suggesting an increase in CVD mortality in individuals undergoing AI treatment when compared to either tamoxifen, a selective oestrogen receptor modulator, or placebo (10, 12, 13). However, most studies were retrospective and uncontrolled.

The exact underlying mechanisms by which AI treatment might confer increased CVD risk are unclear, but the low oestrogen state is postulated to be a contributing factor (15, 16, 17). In non-breast cancer populations, low oestrogen states (18, 19) have been associated with increases in visceral adipose tissue (VAT), the changes which are often mitigated when oestradiol is added back (18, 19). As a bidirectional relationship exists between excess VAT and the many facets of the metabolic syndrome (20), it is possible that severe oestradiol depletion secondary to AI therapy could further the accumulation of VAT. Visceral adipose tissue is both an early marker of increased CVD risk and an important pathogenic mediator of longer-term CVD risk (21); therefore, understanding the potential impact AI therapy has on VAT is important.

We previously reported the results of a prospective cohort study that investigated the effects of 12 months of AI therapy on body composition and various other cardiometabolic risk markers in postmenopausal women with early or locally advanced ER+ breast cancer (22).

We demonstrated that in women initiating AI therapy after 12 months of treatment, the difference between AI and control groups for body composition or other markers of cardiometabolic health was not statistically significant (22).

This study was a pre-planned 12-month extension to our original prospective cohort study. Our hypothesis was that in postmenopausal women newly commencing AIs (without any previous exposure to endocrine therapies), 24 months of AI treatment will be associated with a relevant increase in VAT area. We also anticipated that extending AI treatment for a further 12 months would increase the statistical strength by adding a fourth time point and substantially increasing the number of observations of the study from 267 to 346, as well as a longer time effect for other markers of cardiovascular risk to diverge.

## Methods

We performed a pre-planned 12-month extension of our 12-month prospective cohort study at Austin Health. This extension study was approved by the Austin Health Human Research Ethics Committee (HREC/45582/Austin-2018) and was also preregistered in the original study protocol (before the first participant entered the 12-month prospective study) and is available from the Australian, New Zealand Clinical Trials Registry (identifier ACTRN12619001046190). At the first visit, each participant provided written informed consent for collection of data for a total of 24 months.

Trial methods have been described previously (22). Briefly, participants were screened from our Breast Oncology-Endocrine Multidisciplinary Clinic between April 2019 and September 2020. Participants were recruited as part of the AI cohort if they were postmenopausal women with early stage or locally advanced (stages I–III), ER+ breast cancer, commencing an AI with the intention to continue treatment for a minimum of 12 months or as part of the control cohort if they were postmenopausal women diagnosed with breast pathology within 3 months of screening, not intending to commence endocrine therapy. Anastrozole 1 mg and letrozole 2.5 mg were the type and doses of AIs prescribed at baseline. Exclusion criteria included a history of metastatic or recurrent breast cancer, prior, active or recurrent malignancy within the past 5 years (except non-melanoma skin cancers), exposure to endocrine therapy >8 weeks in the 12 months prior to study screening, exposure to hormone replacement therapy within the 3 months prior to study screening, exposure to glucocorticoid use for ≥2 weeks continuously within the 3 months prior to screening or preexisting diabetes (requiring glucose-lowering medication). Participants were also required to be English speaking for the completion of quality-of-life questionnaires (the results of which will be reported in a future publication). Study visits were conducted at 0, 6, 12 and 24 months.

### Outcome measures

Our pre-specified primary end point for the study was the change in VAT area at 12 months, and these results have previously been published (22). Therefore, data from 24-month time points reflect additional data points of the pre-planned extension. For the extended study, our primary end point remained VAT area. Secondary end points were subcutaneous adipose tissue (SAT) area, total body fat and lean mass. Additional end points were regarded as exploratory but included reactive hyperaemia index (RHI), liver function tests (LFTs), liver fibrosis, liver steatosis and abdominal aortic calcification (AAC).

### Dual x-ray absorptiometry (DXA)

Measurements of body composition were performed using the Hologic Horizon A, DXA scanner (Hologic Inc., Bedford, MA, USA) at baseline and at 6, 12 and 24 months. All procedures were conducted in accordance with the department’s standard Whole-Body protocol for the Hologic Horizon A DXA scanner using the APEX software version 13.6. The software reports total body and regional fat mass and lean mass. Visceral fat is estimated from a subregion of the android region extending 5.2 cm superiorly from the top of the iliac crests. The software estimates the total circumferential subcutaneous fat in this region by extrapolating from measured subcutaneous fat between the skin and abdominal wall musculature on both sides. This estimate of subcutaneous fat is subtracted from the measured total fat in this region to yield an estimate of visceral fat (23, 24). Coefficients of variation for all body composition parameters using this DXA system at our institution were <3% (25).

### Clinical and anthropometric measures

Height and weight were measured at each study visit using standard anthropometric techniques, and measurements were then utilized to calculate the body mass index. Waist circumference (WC) was measured according to the World Health Organization guidelines (26). Blood pressure was measured at baseline and at 6, 12 and 24 months with an aneroid sphygmomanometer in women resting for at least 10 min in a sitting position. Mean arterial blood pressure (MAP) was then estimated.

### Pedometer recordings

Pedometers were provided to record participants’ daily step counts for a week after each study visit. The mean daily step count was calculated for each participant at baseline, 6 , 12 and 24 months. At the end of 24 months, participants were categorized into either a higher activity (mean daily step count ≥ the cohort median for three or more of the four study visits) or lower activity group (mean daily step count < the cohort median for three or more of the four visits) based on their pedometer recordings.

### Blood samples

Fasting venous blood samples were collected at baseline, 3, 6 and 12 months. Fasting glucose (reference range (RR) 3.3–6.0 mmol/L) was measured using a hexokinase photometric assay (Roche Diagnostics) with CVs of 1.5% at 4.8 mmol/L and 15.5 mmol/L. Haemoglobin A1c (HbA1c) (non-diabetic RR < 6.5%) was measured using turbidimetric inhibition immunoassay (Roche Diagnostics) with CVs of 2.7% at 38 mmol/mol and 1.9% at 80 mmol/mol.

Other biochemistry was performed using routine procedures at the biochemistry department, Austin Health.

### EndoPAT

Endothelial function was measured using the US Food and Drug Administration (FDA) approved EndoPAT (endo-PAT2000 version 3.2.4; Itamar Medical Ltd, Caesarea, Israel). The subject was positioned supine, and a pneumatic cuff was placed on the upper arm (study arm). Pneumatic plethysmographic probes were placed on the index finger of each hand and inflated to allow continuous recording of pulsatile blood volume responses. After 5 min, the pneumatic cuff on the study arm was inflated to 200 mmHg or 60 mmHg above systolic pressure for a period of 5 min. The cuff was then deflated to induce reactive hyperaemia, and pulse-volume recordings are continued for 5 further minutes. The reactive hyperaemia index (RHI) was defined as the ratio of the average pulse-volume amplitude over a 1-min time interval (started 1 min after cuff deflation) divided by the average pulse-volume amplitude over a 3.5-min time period before cuff inflation. The value from the study arm was then normalized to the control arm to compensate for potential systemic alterations in vascular tone. An RHI of <1.67 was indicative of peripheral microvascular endothelial dysfunction (27). This test was performed at baseline, 12 and 24 months. RHI was not assessed in women who had bilateral mastectomies and/or axillary dissections.

### FibroScan

Vibration-controlled transient elastography (FibroScan 430, Echosens, Paris, France), an FDA-approved method, was utilized to evaluate hepatic steatosis. FibroScan was performed at both baseline, 12 and 24 months. Controlled attenuation parameter (CAP) measurements using the M probe were conducted, and the final CAP score was reported as the mean of 10 readings. The steatosis grade was then assigned according to the mean CAP score (28). CAP measurements using the M probe have been comparable to liver biopsies in theability to accurately identify and assess liver steatosis (29, 30, 31).

### Abdominal aortic calcification

Lateral spine images for all participants were obtained on the Hologic Horizon A, DXA scanner (Hologic Inc., Bedford, MA, USA) in the supine position for prevalent vertebral fracture assessment (VFA) at 0, 6 and 12 and 24 months. The use of low-dose DXA lateral spine imaging with DXA has been shown to strongly correlate with standard radiography in the detection of AAC (32). A single radiologist (MM) evaluated all images for the AI and control cohorts utilizing the validated AAC-24 scoring system. Details of the AAC-24 have been published elsewhere (33), but briefly, the abdominal aorta is divided into four segments utilizing the L1–L4 lumbar vertebrae as anatomic landmarks. In each of these segments, aortic calcification is scored: 0 if there is no evidence of calcification, 1 if one-third or less of the aortic wall is calcified, 2 if greater than one-third but less than two-thirds is calcified, or 3 if more than two-thirds of the aortic wall is calcified. Scores can therefore range from 0 to 6 for each vertebral level, and total overall AAC score can range from 0 to 24. The radiologist was blinded to the participant cohort status as well as all patient characteristics except for age.

### Medication adjustments

We pre-specified that participants would not be excluded from follow-up if CVD medications were commenced or doses were modified during the study period by non-study clinicians. All medication changes were recorded at each visit.

### Power analysis

Our power analysis for our prespecified 12-month end point has previously been reported and published (22). In Brief, expected changes in VAT were estimated from a retrospective study examining the effects of ≥6 months of AI therapy on VAT (34). We hypothesized that 12 months of AI therapy would result in an increase in VAT area by approximately 25% in the AI group compared to controls. Assuming a standard deviation of 40 cm^2^, a power of 80% and a two-sided significance level of 5%, we would require 80 participants. To allow for 30% attrition, we aimed to recruit a total of 104 participants.

### Statistical analysis

Crude data were reported, along with the derived estimates. To compare characteristics between the groups at the first visit, Wilcoxon rank sum test was used, and data were tabulated as a median and interquartile range. Frequencies were compared with the *χ*2 test and Fisher’s exact test if case numbers were low.

We relied on repeated‐measures mixed‐effects model analysis to assess the treatment effect between intervention vs control group over time (35). The models included, as fixed effects, time points, groups (AIs and control) and the interaction term of time points by group and, as a random effect, at the subject level. Further details have been previously reported together with the 12-month study (22). Sensitivity analysis assessed the influence of additional nonsignificant covariables.

Under the intention-to-treat principle, the mixed model accounted for the joint between-subject and within-subject variation in all included subjects and available data under the missing-at-random assumption of observations at single time points. Outcomes for the treatment effect are reported as mean adjusted difference (MAD) and the profiled 95% confidence interval (CI) of the MAD. The significance level was tested as a single two-sided *P*- value over all time points. A two‐sided *P-*value of <0.05 was considered indicative of statistical significance. Because of the inter‐related outcomes and exploratory nature of other parameters, no adjustments for multiple testing were made. Analyses were performed with the *R* statistical base package (version 4.2.2 for Mac) and the added packages lme4 1.1‐31 and effects 4.2‐2 (35, 36).

## Results

Two-hundred and sixty-six postmenopausal women referred to our Breast Oncology-Endocrine Multidisciplinary Clinic between April 2019 and September 2020 were screened for study eligibility.

**Figure 1:**
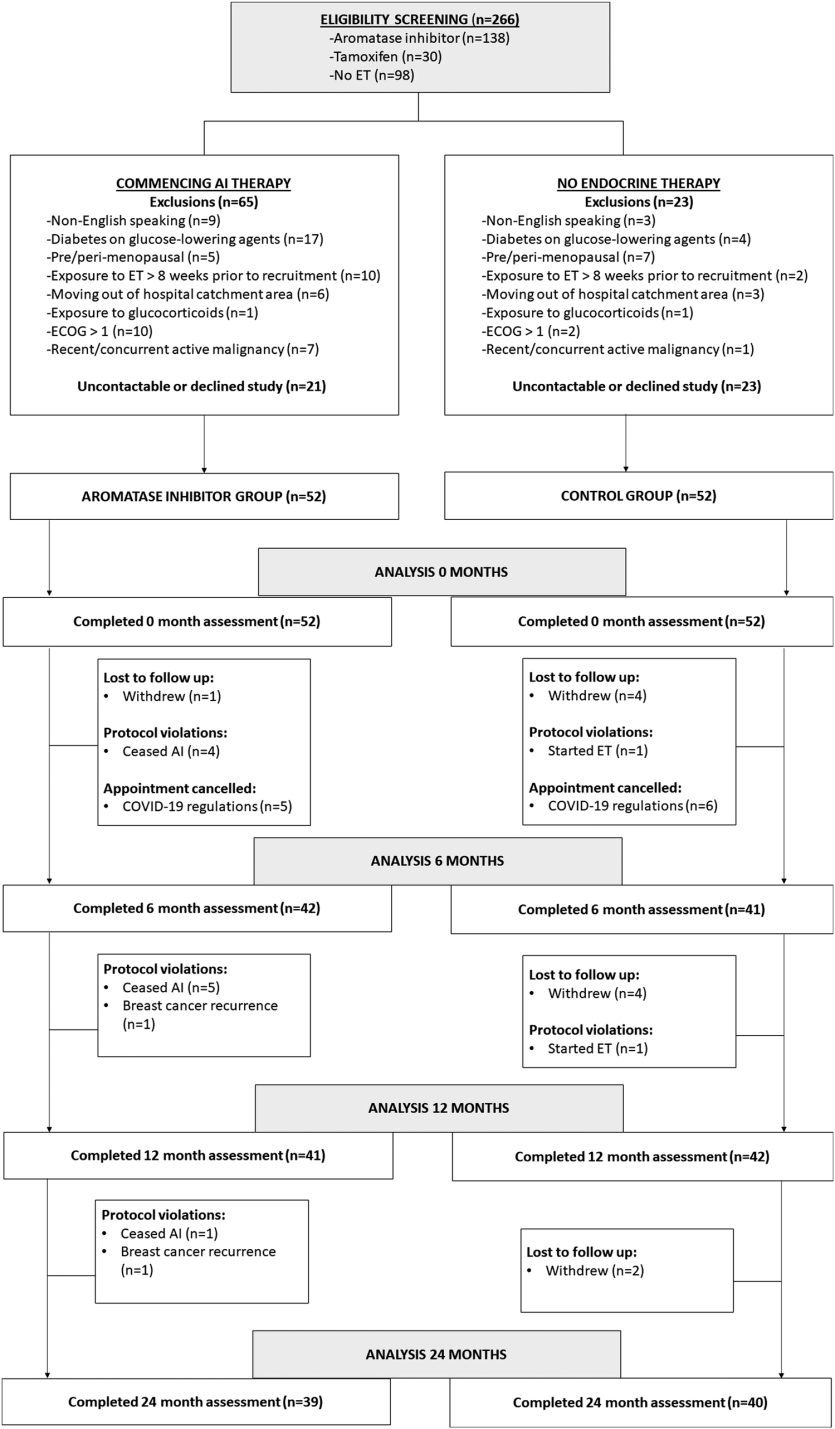
Study flow diagram. AI, aromatase inhibitor; COVID-19, coronavirus disease 2019; ET, endocrine therapy; ECOG, Eastern Cooperation Oncology Group.

A total of 104 women were recruited and enrolled (Fig. 1). For the control group, the mean age was 63.5 years, 33 (63.5%) were Anglo-Saxon and the mean body mass index was 28.6 kg/m^2^. For the AI group, the mean age was 64.5 years, 33(63.5%) were Anglo-Saxon and the mean body mass index was 28.6 kg/m^2^. By design, participants in the control group were more likely to have benign or precancerous breast pathology, whereas all participants in the AI group had breast cancer. Forty-three (82.7%) participants in the AI group had either stage I or II breast cancer, whilst 9 (17.3%) had stage III breast cancer. Women in the control group were less often treated with surgery or radiotherapy compared to the AI group, and no patients in the control group required chemotherapy or trastuzumab therapy (Table 1).

**Table 1:** Participant characteristics at first visit. Data are presented as *n* (%), median (IQR) (22).

	Control group (*n* = 52)	AI group (*n* = 52)	*P*-value
**Age (years)**	63.5 (58.8; 69.0)	64.5 (58.8; 71.0)	0.91
**Ethnicity**			0.90
Anglo-Saxon	33 (63.5)	33 (63.5)	
European	16 (30.8)	14 (26.9)	
Asian	3 (5.8)	4 (7.7)	
Other	0 (0)	1 (1.9)	
**BMI (kg/m^2^)**	28.6 (26.0; 33.0)	28.6 (25.6; 34.1)	0.73
**Breast pathology**			**<0.001**
Early breast cancer	17 (32.7)	43 (82.7)	
Locally advanced breast cancer	0 (0)	9 (17.3)	
DCIS	23 (44.2)	0 (0)	
Other breast pathology^a^	12 (23.1)	0 (0)	
**Breast treatment**			
Aromatase inhibitor	0 (0)	52 (100)	**<0.001**
Anastrozole	0 (0)	26 (50)	
Letrozole	0 (0)	26 (50)	
Surgery	46 (88.5)	52 (100)	**0.03**
Radiotherapy	22 (42.3)	43 (82.7)	**<0.001**
Chemotherapy	0 (0)	15 (28.8)	**<0.001**
Trastuzumab	0 (0)	6 (11.5)	**0.03**
**Smoking status**			0.28
Never	22 (42.3)	29 (55.8)	
Current	5 (9.6)	6 (11.5)	
Ex-smoker	25 (48.1)	17 (32.7)	
**Alcohol intake (STD/day)**			0.65
No intake	28 (53.8)	23 (44.2)	
<1	16 (30.8)	22 (42.3)	
1–2	6 (11.5)	5 (9.6)	
>2	2 (3.9)	2 (3.9)	
**Activity level (steps/day)**	5790 (4030; 7117)	4949 (3197; 8240)	0.92
**ASCVD risk^b^**			0.49
Low	17 (34.0)	18 (35.3)	
Borderline	7 (14.0)	4 (7.8)	
Intermediate	12 (24.0)	18 (35.3)	
High	14 (28.0)	11 (21.6)	
**CVD risk factors**			
Metabolic syndrome^c^	15 (28.8)	17 (32.7)	0.83
CVD	2 (3.9)	2. (3.9)	1.00
Cerebrovascular disease	5 (9.6)	2 (3.9)	0.44
Pre-diabetes or diabetes	3 (5.8)	2 (3.9)	1.00
Hypertension	17 (32.7)	23 (44.2)	0.31
Hypercholesterolaemia	17 (32.7)	13 (25.0)	0.52
Family history of premature CVD	8 (15.4)	9 (17.6)	0.97
Family history of premature cerebrovascular disease	3 (5.8)	3 (5.9)	1.00
Premature menopause	5 (9.6)	6 (11.5)	1.00
**CVD medications**			
Antiplatelet	4 (7.7)	4 (7.7)	1.00
Anticoagulation	2 (3.9)	3 (5.8)	1.00
Antihypertensive	17 (32.7)	23 (44.2)	0.31
Lipid lowering	9 (17.3)	8 (15.4)	1.00
Glucose lowering	0 (0)	0 (0)	1.00
**AAC score**			0.75
0–1	33 (75.0)	30 (78.9)	
2–3	5 (11.4)	5 (13.2)	
>3	6 (13.6)	3 (7.9)	

^a^Other breast pathology included atypical ductal hyperplasia, fibrous tissue, lobular carcinoma *in situ*, microcalcifications and papilloma.

^b^The diagnosis of metabolic syndrome was made based on the USA National Cholesterol Education Program's Adult Treatment Panel III criteria, as revised by the American Heart Association (48).

^c^ ASCVD risk level was assessed using the American College of Cardiology ASCVD Risk Estimator Plus Calculator (49) (individuals with pre-existing CVD or cerebrovascular disease were automatically categorised as high risk).

AAC, abdominal aortic calcification; AI, aromatase inhibitor; ASCVD, atherosclerotic cardiovascular disease; BMI, body mass index; CVD, cardiovascular disease; DCIS, ductal carcinoma insitu; LCIS, luminal carcinoma insitu; STD, standard drinks.

Coronavirus disease 2019 (COVID-19) regulations were implemented at our institution from March 2020 to August 2020 and included the cessation of all trial visits during this period that were deemed non-urgent. Thirty-nine women in the AI group and 40 in the control group completed follow-up for 24 months, respectively. All women assigned to the AI group available for follow-up at 24 months were still actively taking an AI (75% of the original AI cohort).

### Body composition

**Figure 2:**
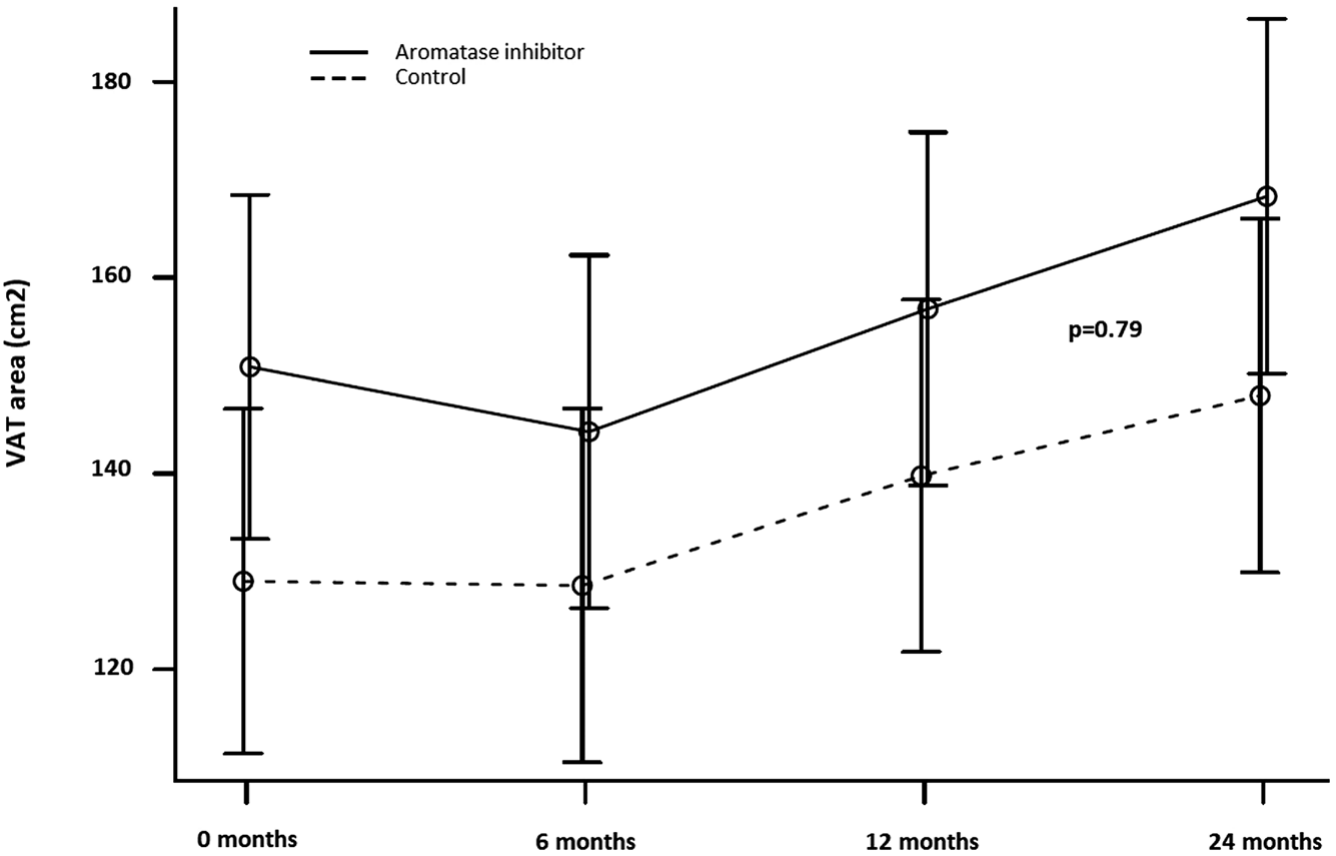
Adjusted mean and 95% CI of VAT area (cm^2^) by group and study visit. The mean-adjusted difference over time between groups, aromatase inhibitor group vs control group is −1.54 cm2 (95% CI: −14.9; 11.9). The significance level was tested as a single *P*-value between groups at all time points (*P* = 0.79).

VAT area did not diverge over time between groups (Fig. 2 and Table 2); the MADs between AI and control groups over 24 months were not statistically significant at any time point (*P* = 0.79), being −1.54 cm^2^ (95% CI: −14.9; 11.9) after 24 months. It should be noted that VAT area increased progressively and significantly over 24 months in the total cohort by 18.1 cm^2^ (95% CI: 11.4; 24.9) from baseline (*P* < 0.001), with the two groups increasing in parallel.

**Table 2:** Body composition and anthropometry.

	Control group^a^ (*n* = 52)	AI group^a^ (*n = 52*)	MAD (AI vs control) (95% CI)	*P*-value^b^
**Primary outcomes VAT (cm^2^)**				0.79
0 months	133 (85.2; 164)	141 (100; 199)		
6 months	124 (82.9; 170)	144 (94.7; 203)	−6.19 (−19.4; 7.07)	
12 months	134 (83.7; 190)	164 (95.1; 227)	−4.84 (−18.0; 8.35)	
24 months	129 (89.3; 190)	150 (106; 242)	−1.54 (−14.9; 11.9)	
**Weight (kg)**				0.84
0 months	75.6 (67.0; 83.9)	74.4 (64.2; 88.6)		
6 months	75.3 (66.1; 82.9)	70.9 (63.2; 88.8)	−0.15 (−1.65; 1.36)	
12 months	74.4 (64.5; 87.6)	75.6 (64.8; 88.3)	−0.62 (−2.13; 0.90)	
24 months	73.1 (65.8; 84.8)	75.0 (62.0; 89.2)	0.02 (−1.52; 1.55)	
**Waist circumference (cm)**				0.36
0 months	96 (84.0, 106)	96 (86.5; 110)		
6 months	96 (85.0; 104)	92 (86.0; 106)	−1.57 (−3.59; 0.46)	
12 months	93 (84.0; 103)	94 (80.5; 106)	−1.09 (−3.15; 0.98)	
24 months	92 (82.8; 102)	94 (82.0; 104)	−1.67 (−3.73; 0.39)	
**Fat mass (kg)**				0.25
0 months	31.8 (27.3; 37.4)	33.2 (25.8; 40.1)		
6 months	32.1 (27.5; 38.1)	32.5 (27.4; 41.7)	−1.35 (−2.67; −0.02)	
12 months	33.5 (27.5; 39.6)	34.7 (26.1; 41.2)	−0.90 (−2.22; 0.41)	
24 months	32.6 (27.9; 40.4)	36.1 (27.4; 42.7)	−0.83 (−2.21; 0.55)	
**Lean mass (kg)**				0.49
0 months	41.0 (36.4; 47.6)	39.8 (35.8; 45.3)		
6 months	39.3 (36.9; 47.1)	40.5 (34.2; 46.3)	0.49 (−0.48; 1.47)	
12 months	39.3 (36.1; 44.9)	40.0 (35.9; 44.6)	0.35 (−0.63; 1.32)	
24 months	38.2 (35.3; 42.6)	38.6 (35.3; 44.1)	0.78 (−0.22; 1.78)	
**SAT (cm^2^)**				0.42
0 months	361 (288; 480)	387 (327; 483)		
6 months	363 (316; 482)	399 (312; 466)	−18.1 (−39.7; 3.6)	
12 months	404 (317; 494)	419 (333; 507)	−5.8 (−27.3; 15.7)	
24 months	377 (334; 502)	437 (320; 510)	−4.1 (−25.9; 17.8)	

^a^Crude values are presented as median (IQR). ^b^The significance level was tested as a single *P*-value between groups over all time points (see section Methods for details).

AI, aromatase inhibitor; MAD, mean adjusted difference; SAT, subcutaneous adipose tissue; VAT, visceral adipose tissue.

Similarly, 24 months of AI therapy did not change total fat mass (MAD −0.83 kg (95% CI: −2.21; 0.55), *P* = 0.25), total lean mass (MAD 0.78 kg (95% CI: −0.22; 1.78), *P* = 0.49) or SAT area (MAD −4.1 cm^2^ (95% CI: −25.9; 17.8), *P* = 0.42), when compared to controls (Table 2).

No statistically significant difference in VAT area was observed between groups after adjusting for activity level category (high vs low) over 24 months, MAD −0.22 cm^2^ (95% CI: −14.8; 14.4), *P* = 0.81.

### Anthropometry

No statistically significant difference in weight and WC was identified between groups over 24 months (MAD 0.02 kg (95% CI: −1.52; 1.55), *P* = 0.84) and (MAD −1.67 cm (95% CI: 82; 104), *P* = 0.36), respectively (Table 2).

### Metabolic markers

There were statistically significant fluctuations in fasting glucose in the AI group when compared to the control group over 24 months (MAD 0.26 mmol/L (95% CI: −0.002; 0.53), *P* = .01). HbA1c levels between groups over 24 months were comparable (MAD 0.10 (95% CI: −0.02; 0.23), *P* = 0.48) (Table 3).

**Table 3:** Metabolic markers.

	Control group^a^ (*n* = 52)	AI group^a^ (*n* = 52)	MAD (AI vs control) (95% CI)	*P*-value^b^
**HbA1c (%)**				
0 months	5.5 (5.3; 5.8)	5.4 (5.2; 5.6)		0.48
3 months	5.5 (5.4; 5.8)	5.5 (5.4; 5.7)	0.03 (−0.09; 0.15)	
6 months	5.6 (5.4; 5.8)	5.6 (5.4; 5.8)	0.08 (−0.04; 0.21)	
12 months	5.6 (5.4; 5.9)	5.5 (5.4; 5.7)	0.04 (−0.09; 0.16)	
24 months	5.5 (5.3; 5.7)	5.5 (5.4; 5.8)	0.10 (−0.02; 0.23)	
**Fasting glucose (mmol/L)**				**0.01**
0 months	5.2 (4.7; 5.4)	5.0 (4.6; 5.5)		
3 months	4.8 (4.3; 5.4)	4.9 (4.5; 5.2)	0.08 (−0.17; 0.32)	
6 months	5.2 (4.7; 5.4)	4.8 (4.3; 5.4)	−0.25 (−0.51; 0.02)	
12 months	5.3 (5.0; 5.5)	5.3 (4.9; 5.7)	−0.01 (−0.27; 0.25)	
24 months	5.2 (4.9; 5.4)	5.4 (5.1; 5.6)	0.26 (−0.002; 0.53)	

^a^Crude values are presented as median (IQR). ^b^The significance level was tested as a single *P*-value between groups over all time points.

AI, aromatase inhibitor; HbA1c, haemoglobin A1c MAD, mean adjusted difference.

### Surrogate markers of cardiometabolic health

No statistically significant difference inMAP (MAD 2.0 mmHg (95% CI: −2.4; 6.3), *P* = 0.44, RHI (MAD 0.40 (95% CI: 0.02; 0.80), *P* = 0.10), AAC (MAD 0.004 (95% CI: −0.58; 0.58), *P* = 0.62), liver steatosis (MAD -13.7 dB/m (95% CI: −35.3; 7.9), *P* = 0.46) and LFTs were identified between groups over 24 months (Table 4).

**Table 4:** Surrogate markers of cardiometabolic health.

	Control group^a^ (*n* = 52)	AI group^a^ (*n* = 52)	MAD (AI vs control) (95% CI)	*P-*value^b^
**Mean arterial pressure (mmHg)**				0.44
0 months	97.0 (91.7; 105)	97.8 (91.8; 103)		
12 months	96.7 (87.2; 106)	95.3 (91.3; 103)	−1.7 (−6.1; 2.6)	
24 months	94.8 (90.2; 104)	102 (95.3; 107)	2.0 (−2.4; 6.3)	
**RHI**				0.10
0 months	2.33 (1.73; 2.94)	2.07 (1.56; 2.70)		
12 months	2.11 (1.50; 2.60)	1.89 (1.56; 2.72)	0.29 (−0.09; 0.67)	
24 months	1.64 (1.48; 1.94)	1.84 (1.54; 2.50)	0.40 (0.02; 0.80)	
**AAC score**				0.62
0 months	0 (0; 1.25)	0 (0; 1.00)		
12 months	0 (0; 2.00)	0 (0; 1.00)	0.25 (−0.32; 0.82)	
24 months	0 (0; 2.00)	0 (0; 2.00)	0.004 (−0.58; 0.58)	
**Steatosis (dB/m)**				0.46
0 months	257 (223; 310)	284 (224; 310)		
12 months	253 (220; 291)	278 (227; 314)	-4.8 (-25.6; 16.1)	
24 months	274 (246; 312)	277 (224; 339)	−13.7 (−35.3; 7.9)	
**ALP (U/L)**				0.73
0 months	85 (68; 102)	84 (71; 105)		
3 months	82 (68; 104)	84 (69; 115)	−3.67 (−14.1; 6.79)	
6 months	79 (69; 95)	87 (66; 109)	−0.08 (−11.2; 11.0)	
12 months	84 (67; 91)	84 (65; 106)	1.70 (−9.39; 12.8)	
24 months	77 (63; 91)	83 (70; 112)	4.36 (−6.87; 15.6)	
**AST (U/L)**				0.64
0 months	23 (20; 29)	24 (20; 27)		
3 months	25 (21; 29)	25 (20; 28)	−1.38 (−3.91; 1.16)	
6 months	24 (21; 31)	25 (20; 30)	−0.58 (−3.24; 2.09)	
12 months	24 (22, 28)	26 (20; 30)	−0.52 (−3.18; 2.14)	
24 months	24 (21; 29)	26 (19; 32)	0.74 (−1.97; 3.45)	
**GGT (U/L)**				0.75
0 months	21 (17–30)	31 (21; 48)		
3 months	22 (16; 26)	31 (22; 46)	−6.00 (−17.6; 5.7)	
6 months	23 (17; 34)	28 (21; 45)	−1.79 (−14.1; 10.5)	
12 months	21 (16; 26)	27 (24; 43)	2.46 (−9.85; 14.8)	
24 months	21 (17; 28)	29 (21; 46)	−1.05 (−13.5; 11.4)	
**ALT (U/L)**				0.76
0 months	20 (16; 28)	23 (19; 31)		
3 months	22 (17; 27)	23 (19; 29)	0.50 (−3.67; 4.67)	
6 months	22 (17; 29)	24 (19; 29)	−0.81 (−5.24; 3.61)	
12 months	20 (17;24)	22 (17; 28)	1.62 (−2.80; 6.03)	
24 months	19 (15; 24)	22 (17; 30)	2.01 (−2.45; 6.48)	

^a^Crude values are presented as median (IQR). ^b^The significance level was tested as a single *P*-value between groups over all time points.

AAC, abdominal aortic calcification; ALT, alanine amino transferase; ALP, alkaline phosphatase; AST, aspartate transaminase AI, aromatase inhibitor; GGT, gamma glutamyl transferase; MAD, mean adjusted difference; RHI, reactive hyperaemia index.

### Post hoc analyses

#### COVID-19 lockdown

During the conduct of our study, the COVID-19 pandemic occurred, followed by state-wide lockdown enforcements. As this could potentially impact upon patients’ activities and thus, their body composition, we analysed and adjusted the outcome at each study visit according to the duration of time spent under lockdown measures. Participants were divided into two categories (lower vs higher proportion of their study period spent in lockdown), using the cohort median time in lockdown (130 days) as the cut-off threshold to define these two groups (22). When adjusting for lockdown category by study visit in the model, this did not improve the model prediction, and the changes in the VAT area remained balanced between the groups over 24 months (MAD −1.62 cm^2^ (95% CI: −15.1, 11.9), *P* = 0.79).

#### Chemotherapy

Fifteen participants in the AI group were also exposed to chemotherapy, which may potentially impact on body composition. We therefore conducted a sensitivity analysis after omitting these 15 participants. No statistically significant difference in VAT area was identified between groups over 24 months (MAD −7.0 cm^2^ (95% CI: −22.0, 8.0), *P* = 0.76).

## Discussion

In postmenopausal women recently commenced on AI therapy, VAT area did not increase over 24 months when compared to controls. Twenty-four months of AI therapy also did not have a substantial negative impact on other measures of body composition, or worsen other cardiometabolic risk markers, when compared to controls.

However, we did note a significant longitudinal worsening of VAT area in both AI and control groups, which occurred in parallel but failed to translate into relevant between-group divergence over time. Whilst the exact clinical implications have not been investigated in this comparative study, these findings do raise concerns for the potential worsening of general CVD risk factors over time, and thus, the need for attentive monitoring and management of increases in VAT as well as other CVD risk factors in this population, irrespective of AI exposure.

### Body composition

Twenty-four months of AI therapy was not associated with an increase in VAT area or other body composition measures when compared to the control group. A robust design and mixed model approach was required to demonstrate that the increases in VAT area run parallel but do not diverge or change between the groups (37). These findings re-emphasize that rigorous controlling is necessary to avoid clinical misinterpretation of study results, wrongly ascribing adverse effects of AI therapy, rather than rightfully acknowledging their non-specific general nature. We specifically note that in using a mixed model and reporting the joint group effects we, unlike previous studies investigating AI-related outcomes, safeguarded against regression to the mean and the frequent ‘difference in nominal significance’ error (37).

Our findings are consistent with our 12-month results, as well as results from a preexisting controlled and relatively short (median 27 months follow-up) study (38). As all other studies in the literature reporting on AI therapy and body composition have either lacked a control group (34), included participants with a prior exposure to tamoxifen, or directly compared AI and tamoxifen populations rather than an endocrine therapy-naive population (39, 40), our data provides important quality evidence to further support the relative cardiovascular safety of AI therapy in the 24 months following initiation.

### Hepatic steatosis and markers of atherosclerosis

Similar to our 12-month results, 24 months of AI therapy did not appear to significantly increase hepatic steatosis when compared to controls. This further supports the notion that in the short-to-medium term at least, AI therapy does not appear to be associated with an increase in hepatic steatosis in postmenopausal women.

In our previous study, 12 months of AI treatment was not associated with adverse effects on RHI when compared to controls. Treatment with AI therapy for an additional 12 months also did not appear to be associated with worse endothelial dysfunction, nor did it appear to be associated with worse AAC scores, when compared to controls. While existing studies investigating the effects of AI therapy on endothelial function report mixed results (41, 42, 43), no current studies have specifically investigated the effects of AI therapy on AAC. Multiple studies have described the utilization of radiographic evidence of AAC, including the use of DXA VFA images, as an independent predictor of incident coronary heart disease, stroke, heart failure and intermittent claudication (44, 45). While our results did not show a statistically significant increase in AAC scores after 24 months of AI treatment when compared to controls, it is important to note that our study cohort had relatively low AAC24 scores at first visit, and therefore likely represented a lower CVD risk population, consistent with the fact that the majority of women within our cohort, at first visit, had low-to-intermediate atherosclerotic CVD risk scores.

Furthermore, atherosclerotic changes have often been described to develop over many years (46). Therefore, it remains possible that 24 months of AI exposure and follow-up may not be sufficient for the development of radiologically apparent changes in AAC.

Longer prospective studies designed to evaluate vascular health as a primary end point are required to validate our findings.

### Glucose metabolism and lipid parameters

As previously described in the 12-month results, our study population displayed significant heterogeneity in glucose homeostasis at first visit (i.e. some women did not have diabetes, whilst a small number had diabetes or pre-diabetes), and glucose-lowering medications were allowed to be introduced or modified during the study (22). This heterogeneity resulted in significant fluctuations in fasting glucose levels taken at a certain point in time and were not matched by HbA1c levels (which were more consistent and comparable between groups). Such heterogeneity also proved to be a significant confounder of the treatment effect of AI on homeostasis model assessment for insulin resistance (HOMA-IR), and a valid MAD for HOMA-IR could, therefore, not be determined. For this reason, we chose not to report on the HOMA-IR 24-month data and cannot comment on a possible diabetogenic effect of AI treatment.

Previous studies have suggested a potential association between AI use and increased incidence of diabetes and insulin resistance in postmenopausal women (38, 47). However, it is unclear if individuals with pre-diabetes were excluded or adjusted for, and, similarly, whether potential confounding caused by glucose-lowering medications was adjusted for. Furthermore, in some studies, the median duration of time exposed to AI therapy for the cohort was not specified. These limitations raise the question of whether baseline heterogeneity in glucose metabolism and uncontrolled changes to glucose-lowering therapy may have confounded the treatment effect reported in these studies, and/or whether a longer duration of exposure to AI therapy must first be exceeded for type 2 diabetes mellitus risk to increase (i.e. >24 months).

Furthermore, in our 12-month results, we were unable to separate the true treatment effect of AI therapy on the various lipid parameters as the initiation and modification of lipid-lowering therapy during the study period was identified to introduce significant confounder. For this reason, we have chosen to not report the results of the 24-month lipid parameters.

### Strengths and limitations

The strengths of this study include its prospective controlled design, its relatively large size, four time points and strength in a number of observations. In addition, its focus is on women newly initiating AI treatment, use of matched controls with breast pathology, exclusion of women pre-treated with tamoxifen and adjustment for confounders such as physical activity and the time spent under COVID-19 lockdown. Also, by extending the study by an additional 12 months, this provides further longitudinal data on the early effects of AI therapy on body composition and cardiometabolic risk. Our statistical design addresses a number of frequent errors that make interpretation of study results less robust and had not been addressed by previous studies. Using mixed models, we were able to rigorously assess the treatment effect, even when both control and AI groups experience non-AI-related changes over time.

Some limitations relevant to this extension study include the controlled but the ethically constrained non-randomized design, participating in the study with the permission to modify CVD medication during the course of the trial, heterogeneity in glucose metabolism and treatment options at first visit and follow-up and the inability to provide oestradiol concentrations as part of our results (22). Given the bias identified as part of the primary 12-month study by lipid-lowering medications as well as the heterogeneity in glucose metabolism at first visit, we decided not to report on the 24-month results for lipids or HOMA-IR.

We acknowledge the exclusion of women with a preexisting diagnosis of diabetes receiving glucose-lowering medication. This was necessary to isolate the effects of AI therapy on VAT and to minimize any potential confounding effects glucose-lowering agents may have on body composition. Also, as the intent of the study was to evaluate the AI treatment effect, we included a control group of women with breast pathology as closely matched to the AI group as ethically possible; we did not include a group of age and body mass index-matched women without breast pathology, and therefore, it is unclear whether longitudinal increases in VAT area observed in participants are related to a diagnosis of breast pathology alone.

Furthermore, whilst 24 months is likely an insufficient follow-up to identify low-dose DXA VFA radiological changes in AAC, other limiting factors may have also contributed to the lack of change observed between AI and control groups. Of note, there was considerable variability in the quality of VFA images amongst our cohort. A number of the VFA images had evidence of poor penetration, incomplete capture of the entire abdominal aorta, and/or the presence of excessive bowel gas, which impacted the radiologist’s ability to confidently report an AAC score. As a consequence, some of these images were considered ‘non-diagnostic’ for AAC and were excluded from further analysis, whilst in others the suboptimal quality may have reduced the sensitivity of AAC detection. It is important to acknowledge, however, that the measurement of AAC on DXA VFA imaging is an ‘opportunistic’ measurement. Therefore, assessment of adequate coverage and image quality of the abdominal aorta would not form part of the routine quality assessment undertaken by the technician. Hence, the described limitations in DXA VFA image quality, in relation to opportunistic ACC scoring, are to be expected and are acceptable.

We also did not collect detailed information regarding each participant’s dietary intake and habits during the study period and acknowledge the impact variations in diet may have on body composition.

Finally, whilst our results did not support our hypothesis, we note the study was not under-powered for the pre-specified outcome measures. As statistically appropriate, we reported profiled CIs to place asymmetric likelihood-base bounds on quantities that were not significantly different from zero to ascertain that the observed non-significant changes were not clinically meaningful.

## Conclusion

In this prospective extension study, newly initiated AI treatment over 24 months was not associated with metabolically adverse changes in body composition nor were significant effects observed in hepatic steatosis or vascular function when compared to controls, in postmenopausal populations. Whilst this is reassuring, the impact of prolonged AI therapy >24 months, on body composition and cardiometabolic health, remains untested. We stress the need for proper controlling, as we observed significant longitudinal change in VAT area for both groups, which occurred in parallel, but failed to translate into relevant between-group divergence over time. The absolute benefits for extended AI therapy should be carefully weighed against the potential risks of longer treatment, in a shared decision-making process between the clinical team and the patient. The worsening of VAT area in both AI and control groups illustrates that CVD risk management should therefore be considered for the general postmenopausal population with breast pathology, over and irrespective of the AI-specific adverse events, which were not confirmed in this study.

## Declaration of interest

MG is on the editorial board of EJE. MG will not be involved in the review or editorial process for this paper on which they are listed as an author. All other authors have nothing to disclose.

## Funding

There is no funding to report relevant to this study. Australian, New Zealand Clinical Trials Registry (identifier ACTRN12619001046190)

## Data availability

The datasets generated during and/or analysed during the current study are available from the corresponding author upon reasonable request.

## Author contribution statement

Material preparation, patient visits and data collection were performed by YC, KV, DW, BH, MR and JH. Participant recruitment was performed by YC, MC, SW, BY and MG. Data preparation and analysis were performed by YC, MM and RH. The first draft of the manuscript was written by YC. YC, RH, JH, MM, AA, MC, SW, BY, JZ and MG had substantial contribution in the conception and design of the manuscript, along with its methodology. All authors have contributed to the drafting and revisions of the manuscript. All authors have given approval for this version of the manuscript to be published.
